# Supplementary material for: Unveiling the Bioactive Potential of the Invasive Jellyfish Phyllorhiza punctata Through Integrative Transcriptomic and Proteomic Analyses
Source: Biomolecules. 2025 Aug 4;15(8):1121. doi: 10.3390/biom15081121 (PMC12383608; doi:10.3390/biom15081121)
Supplement: Supplementary file 1 [file biomolecules-15-01121-s001.zip › Suplementary Material/FileS3/PEPFOLD4/XP_065062749_6842/clusters.html]

 PEP-FOLD clusters


Download archive of all models  here   

|  |  |  |
| --- | --- | --- |
| # | file | sOPEP |
| # cluster 1 - Size: 2 |  |  |
|  | XP\_065062749\_6842-model1 | -26.8543 |
|  | XP\_065062749\_6842-model1.1 | -21.5037 |
| # cluster 2 - Size: 2 |  |  |
|  | XP\_065062749\_6842-model2 | -24.6785 |
|  | XP\_065062749\_6842-model2.1 | -15.5119 |
| # cluster 3 - Size: 3 |  |  |
|  | XP\_065062749\_6842-model3 | -24.4674 |
|  | XP\_065062749\_6842-model3.1 | -17.3187 |
|  | XP\_065062749\_6842-model3.2 | -16.5751 |
| # cluster 4 - Size: 2 |  |  |
|  | XP\_065062749\_6842-model4 | -24.4545 |
|  | XP\_065062749\_6842-model4.1 | -13.4133 |
| # cluster 5 - Size: 2 |  |  |
|  | XP\_065062749\_6842-model5 | -24.1887 |
|  | XP\_065062749\_6842-model5.1 | -16.3656 |
| # cluster 6 - Size: 1 |  |  |
|  | XP\_065062749\_6842-model6 | -23.2449 |
| # cluster 7 - Size: 2 |  |  |
|  | XP\_065062749\_6842-model7 | -23.1508 |
|  | XP\_065062749\_6842-model7.1 | -21.6461 |
| # cluster 8 - Size: 1 |  |  |
|  | XP\_065062749\_6842-model8 | -22.9744 |
| # cluster 9 - Size: 1 |  |  |
|  | XP\_065062749\_6842-model9 | -22.419 |
| # cluster 10 - Size: 2 |  |  |
|  | XP\_065062749\_6842-model10 | -22.1185 |
|  | XP\_065062749\_6842-model10.1 | -19.2649 |
| # cluster 11 - Size: 2 |  |  |
|  | XP\_065062749\_6842-model11 | -21.0833 |
|  | XP\_065062749\_6842-model11.1 | -21.0475 |
| # cluster 12 - Size: 2 |  |  |
|  | XP\_065062749\_6842-model12 | -20.7616 |
|  | XP\_065062749\_6842-model12.1 | -16.9959 |
| # cluster 13 - Size: 3 |  |  |
|  | XP\_065062749\_6842-model13 | -19.802 |
|  | XP\_065062749\_6842-model13.1 | -15.2991 |
|  | XP\_065062749\_6842-model13.2 | -14.7959 |
| # cluster 14 - Size: 2 |  |  |
|  | XP\_065062749\_6842-model14 | -19.5474 |
|  | XP\_065062749\_6842-model14.1 | -11.9559 |
| # cluster 15 - Size: 2 |  |  |
|  | XP\_065062749\_6842-model15 | -19.4853 |
|  | XP\_065062749\_6842-model15.1 | -15.4719 |
| # cluster 16 - Size: 2 |  |  |
|  | XP\_065062749\_6842-model16 | -19.2211 |
|  | XP\_065062749\_6842-model16.1 | -18.9664 |
| # cluster 17 - Size: 4 |  |  |
|  | XP\_065062749\_6842-model17 | -19.1376 |
|  | XP\_065062749\_6842-model17.1 | -18.4778 |
|  | XP\_065062749\_6842-model17.2 | -17.5083 |
|  | XP\_065062749\_6842-model17.3 | -13.4949 |
| # cluster 18 - Size: 1 |  |  |
|  | XP\_065062749\_6842-model18 | -18.6381 |
| # cluster 19 - Size: 1 |  |  |
|  | XP\_065062749\_6842-model19 | -18.0999 |
| # cluster 20 - Size: 3 |  |  |
|  | XP\_065062749\_6842-model20 | -18.0407 |
|  | XP\_065062749\_6842-model20.1 | -16.6929 |
|  | XP\_065062749\_6842-model20.2 | -12.4681 |
| # cluster 21 - Size: 2 |  |  |
|  | XP\_065062749\_6842-model21 | -17.8282 |
|  | XP\_065062749\_6842-model21.1 | -14.7047 |
| # cluster 22 - Size: 2 |  |  |
|  | XP\_065062749\_6842-model22 | -17.6416 |
|  | XP\_065062749\_6842-model22.1 | -15.8719 |
| # cluster 23 - Size: 1 |  |  |
|  | XP\_065062749\_6842-model23 | -17.5019 |
| # cluster 24 - Size: 2 |  |  |
|  | XP\_065062749\_6842-model24 | -17.3333 |
|  | XP\_065062749\_6842-model24.1 | -15.4899 |
| # cluster 25 - Size: 3 |  |  |
|  | XP\_065062749\_6842-model25 | -16.9757 |
|  | XP\_065062749\_6842-model25.1 | -13.6312 |
|  | XP\_065062749\_6842-model25.2 | -10.8895 |
| # cluster 26 - Size: 1 |  |  |
|  | XP\_065062749\_6842-model26 | -16.9641 |
| # cluster 27 - Size: 1 |  |  |
|  | XP\_065062749\_6842-model27 | -16.289 |
| # cluster 28 - Size: 1 |  |  |
|  | XP\_065062749\_6842-model28 | -16.2252 |
| # cluster 29 - Size: 1 |  |  |
|  | XP\_065062749\_6842-model29 | -16.2199 |
| # cluster 30 - Size: 2 |  |  |
|  | XP\_065062749\_6842-model30 | -16.0695 |
|  | XP\_065062749\_6842-model30.1 | -11.5072 |
| # cluster 31 - Size: 1 |  |  |
|  | XP\_065062749\_6842-model31 | -15.8903 |
| # cluster 32 - Size: 2 |  |  |
|  | XP\_065062749\_6842-model32 | -15.8673 |
|  | XP\_065062749\_6842-model32.1 | -12.2832 |
| # cluster 33 - Size: 1 |  |  |
|  | XP\_065062749\_6842-model33 | -15.7262 |
| # cluster 34 - Size: 1 |  |  |
|  | XP\_065062749\_6842-model34 | -15.487 |
| # cluster 35 - Size: 1 |  |  |
|  | XP\_065062749\_6842-model35 | -15.468 |
| # cluster 36 - Size: 1 |  |  |
|  | XP\_065062749\_6842-model36 | -14.8268 |
| # cluster 37 - Size: 1 |  |  |
|  | XP\_065062749\_6842-model37 | -14.7536 |
| # cluster 38 - Size: 1 |  |  |
|  | XP\_065062749\_6842-model38 | -14.0035 |
| # cluster 39 - Size: 4 |  |  |
|  | XP\_065062749\_6842-model39 | -13.8877 |
|  | XP\_065062749\_6842-model39.1 | -13.6941 |
|  | XP\_065062749\_6842-model39.2 | -12.4737 |
|  | XP\_065062749\_6842-model39.3 | -10.9905 |
| # cluster 40 - Size: 2 |  |  |
|  | XP\_065062749\_6842-model40 | -13.8017 |
|  | XP\_065062749\_6842-model40.1 | -12.6025 |
| # cluster 41 - Size: 2 |  |  |
|  | XP\_065062749\_6842-model41 | -13.3694 |
|  | XP\_065062749\_6842-model41.1 | -11.7481 |
| # cluster 42 - Size: 1 |  |  |
|  | XP\_065062749\_6842-model42 | -13.2861 |
| # cluster 43 - Size: 1 |  |  |
|  | XP\_065062749\_6842-model43 | -13.2576 |
| # cluster 44 - Size: 1 |  |  |
|  | XP\_065062749\_6842-model44 | -13.0895 |
| # cluster 45 - Size: 1 |  |  |
|  | XP\_065062749\_6842-model45 | -12.9659 |
| # cluster 46 - Size: 1 |  |  |
|  | XP\_065062749\_6842-model46 | -12.8701 |
| # cluster 47 - Size: 2 |  |  |
|  | XP\_065062749\_6842-model47 | -12.7612 |
|  | XP\_065062749\_6842-model47.1 | -11.9865 |
| # cluster 48 - Size: 1 |  |  |
|  | XP\_065062749\_6842-model48 | -12.4668 |
| # cluster 49 - Size: 3 |  |  |
|  | XP\_065062749\_6842-model49 | -12.3974 |
|  | XP\_065062749\_6842-model49.1 | -12.2802 |
|  | XP\_065062749\_6842-model49.2 | -10.6805 |
| # cluster 50 - Size: 1 |  |  |
|  | XP\_065062749\_6842-model50 | -12.266 |
| # cluster 51 - Size: 1 |  |  |
|  | XP\_065062749\_6842-model51 | -12.2173 |
| # cluster 52 - Size: 1 |  |  |
|  | XP\_065062749\_6842-model52 | -12.1513 |
| # cluster 53 - Size: 2 |  |  |
|  | XP\_065062749\_6842-model53 | -11.6782 |
|  | XP\_065062749\_6842-model53.1 | -10.0641 |
| # cluster 54 - Size: 1 |  |  |
|  | XP\_065062749\_6842-model54 | -11.5976 |
| # cluster 55 - Size: 1 |  |  |
|  | XP\_065062749\_6842-model55 | -11.5869 |
| # cluster 56 - Size: 2 |  |  |
|  | XP\_065062749\_6842-model56 | -11.2726 |
|  | XP\_065062749\_6842-model56.1 | -10.7298 |
| # cluster 57 - Size: 1 |  |  |
|  | XP\_065062749\_6842-model57 | -11.2491 |
| # cluster 58 - Size: 1 |  |  |
|  | XP\_065062749\_6842-model58 | -11.0894 |
| # cluster 59 - Size: 1 |  |  |
|  | XP\_065062749\_6842-model59 | -11.0685 |
| # cluster 60 - Size: 1 |  |  |
|  | XP\_065062749\_6842-model60 | -10.9377 |
| # cluster 61 - Size: 1 |  |  |
|  | XP\_065062749\_6842-model61 | -10.5531 |
| # cluster 62 - Size: 1 |  |  |
|  | XP\_065062749\_6842-model62 | -10.4836 |
| # cluster 63 - Size: 1 |  |  |
|  | XP\_065062749\_6842-model63 | -10.3172 |
|  |  |  |
